# A Comprehensive Approach to Assessment of Testamentary Capacity

**DOI:** 10.3389/fpsyg.2021.789494

**Published:** 2021-12-23

**Authors:** Amanda Kenepp, Ellen Johnson, Grace J. Lee, Preeti Sunderaraman, Natalie L. Denburg, Christopher M. Nguyen

**Affiliations:** ^1^Department of Psychology, Queens College and The Graduate Center (CUNY), New York, NY, United States; ^2^Department of Psychiatry and Behavioral Health, The Ohio State University College of Medicine, Columbus, OH, United States; ^3^Department of Psychology, Ohio University, Athens, OH, United States; ^4^Department of Neurology & The Framingham Heart Study - Brain Aging Program, Boston University School of Medicine, Boston, MA, United States; ^5^Department of Neurology, University of Iowa Carver College of Medicine, Iowa, IA, United States

**Keywords:** aging, financial decision-making, neuropsychological assessment, testamentary capacity, undue influence

## Abstract

The growing aging population raises important implications for legal and clinical systems, including testamentary capacity (TC) assessment. Yet, there are limited comprehensive and standardized assessment measures for TC readily available for clinical use. A review of current assessment methods and standardized approaches for TC assessment is provided. Although several guidelines regarding TC assessment have been proposed in prior literature, existing standardized approaches do not appear to meet full criteria for TC. A comprehensive approach to assessment of testamentary capacity is proposed.

## Introduction

Individuals over the age of 65 are rapidly accounting for a greater proportion of the population than ever before. In fact, the number of individuals aged 65 and older is projected to grow to nearly 1.5 billion worldwide in 2050 (U.S. Department of Health and Human Services, National Institutes of Health, National Institute of Mental Health, 2011). With the improvements in life expectancy and the growing baby-boomer generation reaching retirement age, there will likely be greater focus on end-of-life preparations. Such end-of-life preparations include creating wills and planning the distribution of assets (Peisah et al., 2009; He et al., 2015; Hoffman, 2018). As the next few decades will hold the largest transfer of wealth and assets between generations to date, the execution of an invalid will and testament can have significant financial and interpersonal repercussions (Havens and Schervish, 2003; Marson and Hebert, 2005; Shulman et al., 2005, 2009, 2017; Peisah et al., 2009; Brenkel et al., 2018; Hoffman, 2018). Thus, comprehensive and innovative strategies to determine testamentary capacity (TC) are vital to meet these growing population demands.

## Legal Standards for Testamentary Capacity

The guidelines for assessing TC were initially established with the landmark Banks v. Goodfellow (1870) legal case. Here, the judgment for TC was based on whether an individual could have legal capacity to make a will if he or she suffered from delusions that were unrelated to that will. Outcomes of the case resulted in the development of guidelines that an individual must be free of delusions that could influence the plan for his or her will. Since then, legal interpretations have evolved to include clinical classifications of high, low, or borderline threshold of TC (Marson et al., 2004; Moye, 2005). Complex situations with a large transfer of wealth with multiple possible beneficiaries require a higher threshold of TC due to the greater financial consequences (Shulman et al., 2005, 2007, 2009, 2017; Mart, 2016; Hoffman, 2018). In contrast, the legal standard for TC is lower for less complex situations in order to preserve a person’s right to distribute their assets upon death (Peisah et al., 2009; Shulman et al., 2009; Hoffman, 2018). The court ultimately makes the final decision about TC based on findings and recommendations from clinical assessments (Moye, 2005; Shulman et al., 2007, 2009, 2021; Peisah et al., 2009; Zago and Bolognini, 2020). TC assessments are conducted by legal and/or clinical professionals to address a variety of needs, such as when a testator asks for it, when the legal personnel suspect incapacity, when there are allegations of incapacity, when TC is expected to be challenged in the future, or in cases of retrospective assessment (Marson and Hebert, 2005; Shulman et al., 2007, 2017, 2021; Zago and Bolognini, 2020).

## Undue Influence and Testamentary Capacity

The evaluation of undue influence is critical to the assessment of TC as its presence can invalidate a will. Undue influence is defined as the manipulation of a testator to execute a will that diverges from his wishes (Marson and Hebert, 2005; Peisah et al., 2009). A person is more likely to be subject to undue influence if that person is socially or physically isolated, disabled, subject to family dispute, subject to abuse, grieving the loss of a loved one, or if the person is dependent on others for finances, activities of daily living, or decision-making (Marson and Hebert, 2005; Peisah et al., 2009). Furthermore, undue influence occurs on a spectrum and is dependent on the TC status of an individual. In a person with diminished intellectual or cognitive abilities, very little influence may be needed. Conversely, in a person who is high functioning, more severe manipulation would be necessary to result in undue influence (Marson and Hebert, 2005; Peisah et al., 2009; Shulman et al., 2017; Hoffman, 2018). An individual with certain dementia syndromes may suspect abandonment by the caretaker and could thus be susceptible to undue influence, for example, by having other beneficiaries push for dramatic changes in the will in their favor. On the other hand, a person can appear to be independent and still be susceptible to undue influence which could invalidate his or her will (Marson and Hebert, 2005; Shulman et al., 2005, 2007, 2017; Peisah et al., 2009).

## Clinical Standards for Testamentary Capacity

Existing legal and clinical guidelines have attempted to identify specific skills needed in order to establish TC. One important set of skills needed for establishing TC relate to cognitive functioning. Prior literature suggests that individuals must demonstrate intact cognitive abilities, including memory and executive functioning (Shulman et al., 2007; Hoffman, 2018). In addition, there must be evidence supporting the patient’s awareness of and ability to objectively make financial decisions (Shulman et al., 2007, 2021; Sunderaraman and Cosentino, 2017; Sunderaraman et al., 2018, 2020; Zago and Bolognini, 2020).

Several different components of memory should be evaluated in a TC assessment. At a basic level, a testator should demonstrate comprehension and semantic memory of information pertaining to a will (Marson and Hebert, 2005; Shulman et al., 2007, 2009, 2017; Mart, 2016; Brenkel et al., 2018). Although some guidelines have proposed that the testator may be permitted to use legal documents and be prompted when analyzing their assets (Mart, 2016), others assert that only general knowledge of assets and their value is needed as long as the testator has a clear plan for the will (Shulman et al., 2009, 2017). Under these recommendations, a person must be able to identify what type of asset he has (e.g., real estate property) and be able to approximately identify or categorize its worth (Shulman et al., 2017). TC assessments should also determine if the testator has intact historical and episodic memory in order to ensure that they know who could inherit their property, money, and assets (Marson and Hebert, 2005; Shulman et al., 2007, 2009, 2017; Mart, 2016; Brenkel et al., 2018).

It has also been recommended that TC assessments also determine whether the testator has adequate executive functioning skills to plan for and comprehend the consequences of their actions. For example, assessors should inquire into the testator’s understanding of family relationships and conflicts that may affect the plan for the will (Marson et al., 2004; Moye, 2005; Shulman et al., 2007, 2017; Mart, 2016). The testator should also demonstrate understanding of the consequences that may result from leaving out specific possible beneficiaries (Shulman et al., 2017). Such executive functioning skills would help testators make plans for the creation of their wills and the distribution of their assets (Marson and Hebert, 2005; Shulman et al., 2007, 2009, 2017; Mart, 2016; Brenkel et al., 2018).

Psychiatric concerns should additionally be assessed to determine TC. The extent to which existing neurological and psychiatric conditions could interfere with the testator’s ability to make a will must be determined, particularly related to cognitive decline, delusions, and paranoia (Marson and Hebert, 2005; Shulman et al., 2007, 2009, 2017, 2021; Mart, 2016; Brenkel et al., 2018; Zago and Bolognini, 2020). The existence of psychiatric concerns, such as delusions, does not preclude a testator from having TC and, as such, must be carefully evaluated. Specifically, if delusions do not influence the testator’s opinion of his or her beneficiaries or decision-making regarding the will, TC may be legally granted (Shulman et al., 2009, 2017; Mart, 2016). Diagnoses that may involve impairments in either or both psychiatric and cognitive aspects of functioning, and that may interfere with testamentary capacity include (e.g., traumatic brain injury, Alzheimer disease, frontotemporal dementia, Parkinson’s disease, Huntington’s disease, and motor neuron diseases, such as amyotrophic lateral sclerosis). Other psychiatric and developmental disorders may result in vulnerability to undue influence and decreased judgment when creating a will (e.g., major depressive disorder, bipolar disorder, schizophrenia, and intellectual disability). Notably, many of these diagnoses can occur in younger adults and some result in premature death, but many publications on TC assessment focus on older adults only and especially on dementia. As the demand for TC assessments grows, the need for qualified health care professionals to assess TC also increases (Kaufmann, 2016). However, there are few clinicians who are specialized in TC assessment (Shulman et al., 2009). Furthermore, as there is no standardized assessment for TC, clinicians often rely on proxy methods to assess TC, such as cognitive screening instruments, neuropsychological assessments, and forensic assessment instruments.

## Methods of Testamentary Capacity Assessment

Clinicians typically compile information from a variety of sources to determine if a person has TC. The most common methods for assessing cognitive impairments associated with TC involve the use of brief mental status measures and neuropsychological assessments. Forensic assessment instruments and retrospective TC assessment have also been used to obtain supporting information about a testator’s situation. Here we review the literature on these current approaches.

### Cognitive Assessment

Clinical assessment of TC usually involves a brief screening of cognitive functioning (Shulman et al., 2007; Kaufmann, 2016). More comprehensive neuropsychological evaluations have occasionally been utilized to support assessments of TC (Marson and Hebert, 2005; Sousa et al., 2014; Mart, 2016; Shulman et al., 2017; Brenkel et al., 2018). Specifically for the assessment of TC, the emphasis is often placed on determination of executive functioning, such as judgment, reasoning, and decision-making (Shulman et al., 2017). Although the procedures described above provide valuable information for TC assessment, a general neuropsychological assessment alone is not sufficient for several reasons (Marson and Hebert, 2005; Moye, 2005; Shulman et al., 2009; Sousa et al., 2014). First, neuropsychological assessment cannot account for complexities in a testator’s unique situation, such as family conflict, witness testimony, and number of assets that can influence the interpretation of TC (Shulman et al., 2009; Kaufmann, 2016). Furthermore, a comprehensive assessment must be able to incorporate the evaluation of both legal and clinical concepts of TC. (Shulman et al., 2007; Brenkel et al., 2018).

### Forensic Assessment Instruments

*S*emi-structured interviews have been designed to assess a specific mental capacity in legal settings. For example, the Contemporaneous Assessment Instrument (CAI; Brenkel et al., 2018) is a type of interview that involves the testator and his or her collateral, such as friends, family, or long-term care providers. The CAI involves the review of evidence, such as medical records or related correspondences not specified in previous wills, legal documents, and witness testimony. While considering all the evidence, the clinician makes a judgment of the testator’s TC. The strength of the CAI lies in its ability to consider a comprehensive set of factors that can influence TC. Nevertheless, despite the fact that standardized semi-structured interview procedures exist for other medical purposes (e.g., medical consent; Brenkel et al., 2018) there are no studies to date that focus on the development of a forensic assessment instrument specific to clinical assessment of TC (Sousa et al., 2014; Papageorgiou et al., 2018).

### Retrospective Testamentary Capacity Assessment

Retrospective TC assessment occurs when there is a challenge to the will after it has been executed. It requires a medical or psychological expert to review medical records, test results, witness testimony, correspondence, and other evidence to decide if a testator had sufficient TC at the time of the creation of the will (Marson and Hebert, 2005; Shulman et al., 2007, 2021; Peisah et al., 2009; Zago and Bolognini, 2020). Comprehensive guidelines have recently been published to improve the validity of retrospective assessment of TC (Zago and Bolognini, 2020; Shulman et al., 2021). However, it is more reliable and valid to measure TC contemporaneously than retrospectively due to the variation in neuropsychological capacity over time (Folstein et al., 1975; Marson and Hebert, 2005; Brenkel et al., 2018). Contemporaneous assessment reduces financial and legal burden through avoiding future challenges to the will and reducing the number of cases that go to trial (Shulman et al., 2009; Brenkel et al., 2018). Therefore, establishing a standardized assessment of TC will encourage legal professionals to seek out TC assessment contemporaneously to avoid future will contests and preserve the will’s validity.

## Standardized Approaches of Testamentary Capacity Assessment

Several standardized approaches for evaluating TC currently exist: the Testamentary Capacity Instrument (TCI; Marson and Hebert, 2005) the Legal Capacity Questionnaire (LCQ; Marson et al., 2004) and the Testamentary Capacity Assessment Tool (TCAT; Papageorgiou et al., 2018). The TCI was developed to assess the four criteria established from Banks v. Goodfellow (1870): understanding a will, the extent of assets, all potential heirs, and the plan for the will (Marson et al., 2004; Marson and Hebert, 2005). This instrument also includes a brief evaluation of undue influence, delusions, and hallucinations. It is intended to be supported by general comprehensive neuropsychological assessment (Marson et al., 2004; Marson and Hebert, 2005). The TCI is advantageous in its ability to distinguish if a testator can understand the TC criteria upon recall or recognition of it (Marson et al., 2004). Another advantage of this tool is that results are reported on a spectrum that is beneficial for cases with undue influence (Marson et al., 2004). Although the TCI is a promising instrument, it is not readily accessible for clinical TC assessment.

The LCQ is a screening measurement created specifically for TC assessment (Marson et al., 2004). The LCQ is a first step for legal professionals to screen for possible deficiencies in TC in order to determine whether it is necessary to seek out professional assessment (Marson et al., 2004). The LCQ consists of true or false, multiple-choice, and open-ended questions to inquire of a patient’s basic financial knowledge and understanding of a will and categorizes that person into high, borderline, or low functioning (Marson et al., 2004). Indeed, the LCQ is merely a screening measure, and it has no additional data to support its validity or reliability; thus, it cannot replace formal TC assessment (Marson et al., 2004).

The TCAT (Papageorgious et al., 2018) was recently developed as a standardized measure to determine if a testator needs further forensic assessment. The TCAT includes four sections that assess behavior, intention, judgment, and memory functioning. The testator is tasked with reporting verifiable personal and family information and general financial knowledge, and answers can be scored in a standardized way. The TCAT also includes a self-reported measure of mood to assess for depressive symptoms. Although the tool can be administered by a non-expert, the authors of the TCAT noted that further assessment by a clinical expert is often needed for complex cases in which there are family disputes, a large amount of assets to be distributed, and vulnerability to undue influence. The TCAT has only been validated among older adults with dementia (Papageorgious et al., 2018).

## A Call for a Comprehensive Assessment of Testamentary Capacity

TC is a dynamic construct and clinical decision-making regarding TC must integrate consideration of functional capacities, situational demands, and individual contextual factors. A more comprehensive and standardized TC measure would have clinical utility and provide clear-cut interpretation for legal standards. However, current assessment procedures predominantly rely heavily on the use of clinical judgment when determining TC. While clinical judgment is a valuable tool in decision-making, it is not infallible, and that judgment based on objective, validated data is consistently more accurate than that based on judgment alone (Grove et al., 2000; Ægisdóttir et al., 2006). Accuracy of clinical judgment that can influence case conceptualization includes factors specific to the clinician, such as experience, confidence, and internal biases (Folstein et al., 1975; Grove et al., 2000; Shulman, 2000; Ægisdóttir et al., 2006; Spengler, 2013; Sousa et al., 2014; Miller et al., 2015; Spengler and Pilipis, 2015; Demakis, 2016; Kaufmann, 2016; Sunderaraman and Cosentino, 2017; Sunderaraman et al., 2018, 2020). As the accuracy of clinical judgment is greatly improved with the integration of objective performance measures, best practices related to legal assessment and assessment of cognitive decline require integration of appropriate standardized tools in decision-making (American Psychological Association, 2012, 2013).

In order to cover all required Banks v. Goodfellow (1870) and clinical criteria for TC, a comprehensive assessment of TC should include assessment of neuropsychological functioning, financial knowledge, potential for undue influence, and knowledge of testamentary. A more comprehensive and standardized TC measure would have clinical utility and provide clear-cut interpretation for legal standards. Here, we present recommendations for a comprehensive TC assessment that includes examining aspects of cognition, financial capacity, knowledge of testamentary, mood and psychiatric factors, and presence of undue influence.

### Cognitive Assessment

A neuropsychology battery consisting of tests in the domains of language, memory, and executive functioning is necessary to examine the cognitive functioning related to TC. Specifically, tests evaluating verbal language abilities (e.g., comprehension, abstraction, expressive, and receptive language skills) are critical for the understanding the purposes and consequences of a will. Examining memory functioning, such as episodic long-term and short-term memory abilities, is critical to evaluate the person’s ability to recall recent changes in the personal circumstances that impacts distribution of possessions. Lastly, intact executive functioning is necessary for one’s ability to plan and distribute property and assets during the creation of a will.

### Financial Decision-Making Assessment

The instruments to assess financial capability typically use either interviews, informant reports, performance-based items, or a combination of these approaches (National Academies of Sciences, Engineering, and Medicine, 2016). Some of these instruments are stand-alone assessments or used in combination with other functional assessments (National Academies of Sciences, Engineering, and Medicine, 2016). The use of instruments that rely on self- or informant reports is subjective and prone to biases, such as under- or over-estimation of one’s abilities (Ponsford et al., 2000; Martin et al., 2012; Sunderaraman et al., 2019). Moreover, subjective reports cannot be easily verified for accuracy, especially for those with diminished cognition (Sunderaraman et al., 2019). Finally, impaired self-awareness of one’s financial ability can be even more dangerous as it can influence the information collected *via* subjective report and lead to suboptimal decisions and behaviors (Sunderaraman et al., 2020). Therefore, it is suggested that financial capability should preferably be evaluated in an objective manner using performance-based tasks. Depending on the age and experience of the individual, traditional and modern, online-based approaches can be used to evaluate financial abilities (Sunderaraman et al., 2020).

### Knowledge of Testamentary

An assessment of testamentary knowledge can satisfy Banks v. Goodfellow’s criteria of evaluation for the knowledge of what one’s assets are, knowledge of who can inherit those assets, and the ability to make a clear plan for the distribution of assets. For example, increasingly complex vignette-based hypothetical scenarios will be used to assess the individual’s understanding of the act of making a will and its effects. In these scenarios, the individual can be asked open-ended questions about the hypothetical scenarios to determine who might be expected to benefit from his or her estate and explain any significant conflict in the context of the testator’s life situation (Nguyen et al., 2019).

### Mood and Psychiatric Factors

The evaluation of emotional functioning (i.e., cognitive and psychiatric assessments) is necessary to identify and rule out evidence of severe cognitive impairment, psychosis, and/or the presence of delusions that could influence testamentary capacity. This process of the assessment will include behavioral observations, chart review, and clinical interview.

### Presence of Undue Influence Subscale

This aspect of the comprehensive TC assessment should assess for the presence of undue influence using Yes/No questions, (e.g., “Is this your idea?” and “Did you feel pressure to do this?”). These questions will assess whether the individual had an active participation in the development of the will, if there are recent changes to the will, if the changes were expected, and if there are outside individuals that contributed to changes against the individual’s wishes.

In summary, the proposed assessment methodology for TC would expand upon limitations of current methodologies and allow for enhanced ease of integration through the inclusion of a comprehensive approach to evaluate each element of TC. This would enable clinicians to categorize a person into varying levels of capacity based on each domain. Thus, a comprehensive TC assessment can help convey the whole picture of each individual and inform the clinician if the individual is able to compensate for deficiencies in one area through strengths in another. A more flexible model of legal capacity measurement can also preserve the autonomy of individuals undergoing TC evaluation (Committee on the Rights of Persons with Disabilities, 2014; Sabbata, 2020). Furthermore, a comprehensive standardized assessment battery with the potential to be well-validated would increase consistency across TC evaluations and reduce bias in decision-making due to variability in factors that influence clinical judgment.

## Data Availability Statement

The original contributions presented in the study are included in the article/supplementary material, further inquiries can be directed to the corresponding author.

## Author Contributions

AK, ND, and CN contributed to conception and design of manuscript. AK and CN wrote the first draft of the manuscript. EJ, GL, and PS wrote sections of the manuscript. All authors contributed to manuscript revision, read, and approved the submitted version.

## Funding

This study was funded by the American Psychological Association Society for Clinical Neuropsychology (Division 40) Early Career Pilot Study Awards to CN.

## Conflict of Interest

The authors declare that the research was conducted in the absence of any commercial or financial relationships that could be construed as a potential conflict of interest.

## Publisher’s Note

All claims expressed in this article are solely those of the authors and do not necessarily represent those of their affiliated organizations, or those of the publisher, the editors and the reviewers. Any product that may be evaluated in this article, or claim that may be made by its manufacturer, is not guaranteed or endorsed by the publisher.
